# Virulence and Antibiotic Resistance Genes in *Listeria monocytogenes* Strains Isolated From Ready-to-Eat Foods in Chile

**DOI:** 10.3389/fmicb.2021.796040

**Published:** 2022-02-21

**Authors:** Julio Parra-Flores, Ondrej Holý, Fernanda Bustamante, Sarah Lepuschitz, Ariane Pietzka, Alejandra Contreras-Fernández, Claudia Castillo, Catalina Ovalle, María Paula Alarcón-Lavín, Ariadnna Cruz-Córdova, Juan Xicohtencatl-Cortes, Jetsi Mancilla-Rojano, Miriam Troncoso, Guillermo Figueroa, Werner Ruppitsch

**Affiliations:** ^1^Department of Nutrition and Public Health, Universidad del Bío-Bío, Chillán, Chile; ^2^Science and Research Centre, Faculty of Health Sciences, Palacký University Olomouc, Olomouc, Czechia; ^3^Environmental and Public Health Laboratory, Regional Secretariat of the Ministry of Health in Maule, Talca, Chile; ^4^Austrian Agency for Health and Food Safety, Institute for Medical Microbiology and Hygiene, Vienna, Austria; ^5^Food Quality Testing and Certification Laboratory, Universidad del Bío-Bío, Chillán, Chile; ^6^School of Nutrition and Dietetics, Universidad del Bío-Bío, Chillán, Chile; ^7^Intestinal Bacteriology Research Laboratory, Hospital Infantil de México Federico Gómez, Mexico City, Mexico; ^8^Faculty of Medicine, Biological Sciences Graduate Program, Universidad Nacional Autónoma de México, Mexico City, Mexico; ^9^Microbiology and Probiotics Laboratory, Institute of Nutrition and Food Technology, Universidad de Chile, Santiago, Chile

**Keywords:** *Listeria monocytogenes*, ready-to-eat foods, virulence, resistance genes, whole-genome sequencing, CRISPR-Cas

## Abstract

*Listeria monocytogenes* is causing listeriosis, a rare but severe foodborne infection. Listeriosis affects pregnant women, newborns, older adults, and immunocompromised individuals. Ready-to-eat (RTE) foods are the most common sources of transmission of the pathogen This study explored the virulence factors and antibiotic resistance in *L. monocytogenes* strains isolated from ready-to-eat (RTE) foods through *in vitro* and *in silico* testing by whole-genome sequencing (WGS). The overall positivity of *L. monocytogenes* in RTE food samples was 3.1% and 14 strains were isolated. *L. monocytogenes* ST8, ST2763, ST1, ST3, ST5, ST7, ST9, ST14, ST193, and ST451 sequence types were identified by average nucleotide identity, ribosomal multilocus sequence typing (rMLST), and core genome MLST. Seven isolates had serotype 1/2a, five 1/2b, one 4b, and one 1/2c. Three strains exhibited *in vitro* resistance to ampicillin and 100% of the strains carried the *fosX*, *lin*, *norB*, *mprF*, *tetA*, and *tetC* resistance genes. In addition, the *arsBC*, *bcrBC*, and *clpL* genes were detected, which conferred resistance to stress and disinfectants. All strains harbored *hlyA*, *prfA*, and *inlA* genes almost thirty-two the showed the *bsh, clpCEP, hly, hpt, iap/cwhA, inlA, inlB, ipeA, lspA, mpl, plcA, pclB, oat, pdgA*, and *prfA* genes. One isolate exhibited a type 11 premature stop codon (PMSC) in the *inlA* gene and another isolate a new mutation (deletion of A in position 819). The Inc18(rep25), Inc18(rep26), and N1011A plasmids and MGEs were found in nine isolates. Ten isolates showed CAS-Type II-B systems; in addition, Anti-CRISPR AcrIIA1 and AcrIIA3 phage-associated systems were detected in three genomes. These virulence and antibiotic resistance traits in the strains isolated in the RTE foods indicate a potential public health risk for consumers.

## Introduction

Ready-to-eat (RTE) foods are defined as any food in a raw state or one that is handled, processed, mixed, cooked, or prepared and is consumed without any further processing (Monteiro, 2010). The RTE foods are a practical alternative to meet daily food needs; however, they are not exempt from contamination by biological hazards such as *Salmonella* spp., pathogenic *Escherichia coli*, and *Listeria monocytogenes* (Becker et al., 2019).

*Listeria monocytogenes* is a Gram-positive facultative anaerobic, ubiquitous, and persistent bacterium in food processing plants. Due to inadequate hygiene and manufacturing practices, this pathogen contaminates foods such as fresh or frozen fruits and vegetables, unpasteurized dairy products, sausages, and fish (Amajoud et al., 2018). Cheeses, sausages, meats, and fish are the most frequently associated with outbreaks of this pathogen worldwide (Kurpas et al., 2018; Ryser, 2021). *L. monocytogenes* causes listeriosis, a disease characterized by low morbidity but high mortality in those who are infected, and the most at risk groups are pregnant women, newborns, children, and older adults (Schlech, 2019). In Europe and North America, invasive listeriosis affects 0.3–0.6 persons per 100,000 inhabitants annually (Maertens de Noordhout et al., 2017; European Centre for Disease Prevention and Control [ECDC], 2018). In Chile, *L. monocytogenes* has been under mandatory laboratory notification and surveillance since 2005 (Bustamante et al., 2020). There were 97 cases in 2018 and 69 in 2019 with lethality of 22% and 26%, respectively (MINSAL: Ministerio de Salud Chile, Departamento de Estadísticas e Información en Salud [DEIS], 2019). Adults aged 65 and older and pregnant women were the most affected groups. Pregnancy was terminated in 50% of cases and abortion or fetal death was reported in 21% (MINSAL: Ministerio de Salud Chile, Departamento de Estadísticas e Información en Salud [DEIS], 2019). The RTE foods were the main source of infection associated with these listeriosis cases (MINSAL: Ministerio de Salud Chile, Departamento de Estadísticas e Información en Salud [DEIS], 2019).

The severity of *L. monocytogenes* infection is associated with several factors such as infecting dose, host immunity, and expression of virulence factors (adherence, invasion, immune modulator, intracellular survival, toxins, and mobile genetic elements), and the presence of CRISPR-Cas as a virulence regulator (Falavina dos Reis et al., 2011; Louwen et al., 2014; Pouillot et al., 2016; Buchanan et al., 2017; Kwon et al., 2020). In addition, there are other factors such as resistance to disinfectants and antibiotics, especially beta-lactams (Olaimat et al., 2018). The capacity to resist to adverse environmental conditions (heat and cold stress) allow the persistence and colonization throughout the food chain by forming contamination reservoirs that are difficult to control (Bolocan et al., 2016; Bucur et al., 2018). The *Listeria* species can be categorized into different serotypes according to the serological reactions of the listerial somatic antigen (O-antigen) and flagellar antigen (H-antigen) with specific antisera. *L. monocytogenes* can be classified into at least 13 serotypes (Orsi et al., 2011), three of them (1/2a, 1/2b, and 4b) are involved in over 95% of human clinical cases, and serotype 4b exhibits the strongest epidemiological association with human listeriosis (Maury et al., 2016; Lee et al., 2018).

Whole-genome sequencing (WGS) currently allows *in silico* generation of a wealth of information about pathogenic strains, including a more precise description of the taxonomic differences and similarities between them. The WGS technology is used to more precisely identify the pathogen and genotype it by multilocus sequence typing (MLST), clonal complex (CC) determination, core genome MLST (cgMLST), CRISPR-Cas, and serogrouping. WGS also enables the detection of antibiotic resistance and virulence genes, plasmids, and mobile genetic elements (MGEs); this information provides a more precise epidemiological relationship (Leopold et al., 2014; Ruppitsch et al., 2015a; Moura et al., 2017; Hurley et al., 2019; Kwon et al., 2020; Stessl et al., 2021). The use of WGS has been fundamental in the successful investigation of recent extensive outbreaks of *L. monocytogenes* in South Africa (2017–2018) and Germany (2018–2019) (Allam et al., 2018; Halbedel et al., 2020).

According to Chilean health authorities, RTE foods are the main source of infection associated with cases of this disease in Chile (MINSAL: Ministerio de Salud Chile, Departamento de Estadísticas e Información en Salud [DEIS], 2019). However, information about the diversity, pathogenicity, and virulence of *L. monocytogenes* in Chile is still limited and incomplete. Our study contributes to a better understanding of *L. monocytogenes* with respect to genotype diversity, virulence, antibiotic resistance, and cas genes by generating necessary and indispensable scientific evidence. Many of the evaluated foods in this study are marketed in the Americas. Therefore, given the need for updated information on this pathogen in Chile, we studied virulence factors and antibiotic resistance in *L. monocytogenes* strains isolated from RTE foods by *in vitro* and *in silico* testing using whole-genome sequencing (WGS).

## Materials and Methods

### Samples

A total of 436 samples of retail RTE foods were analyzed; these are regarded as at risk foods sampled as part of sampling plan the Emerging Pathogens Program of the Health Authority in the Maule Region, Chile, and which are regulated by the Chilean Food Sanitary Regulations (RSA). Samples used for our study consisted of cheeses (*n* = 161), cooked meats (artisanal ham, pâté, sausages, and blood sausage) (*n* = 186), pre-processed fruits and vegetables (chopped fruit, fruit salads with strawberries, melon, and peaches, and leafy vegetable salads) (*n* = 22), and meals and mixed dishes with raw and/or cooked ingredients (*n* = 67).

### Isolation of *Listeria monocytogenes*

Isolation was performed on the basis of the ISO 11290–1:2017 standard. Each 25 g food sample was inoculated in 225 mL half Fraser broth (Oxoid, Basingstoke, United Kingdom) as primary selective enrichment and homogenized in a stomacher (Seward 400, Radnor, PA, United States). Incubation was performed at 30 ± 1°C for 25 ± 1 h; the second enrichment consisted of 0.1 mL of the broth culture inoculated in 10 mL of full-strength Fraser broth, which was cultured at 37°C for 24 ± 2 h. A loopful of each of the half- and full-strength Fraser broths were plated on the *Listeria* chromogenic agar base according to Ottaviani and Agosti (ALOA) (Merck, Darmstadt, Germany). These plates were incubated at 37°C for 24–48 h. Five typical colonies from each ALOA agar plate were restreaked on tryptic soy agar supplemented with 0.6% yeast extract (TSA-YE) (Sigma, Darmstadt, Germany) as a non-selective medium, and these were incubated at 37°C for 24–48 h. Colonies from the TSA-YE were verified by Gram staining, catalase reactions, oxidase tests, carbohydrate utilization test, CAMP tests, and motility at 20–25°C. These colonies were stored for further study.

### Detection of *Listeria monocytogenes*

Detection of *L. monocytogenes* was performed with the Vitek Immunodiagnostic Assay System (VIDAS) (bioMerieux Vitek Inc., Hazelwood, MO, United States) according to the manufacturer’s instructions. The equipment automatically measured and interpreted data, reporting detection as positive or negative according to the validated AOAC (Official Method of Analysis No. 2004.2) protocol for food matrices.

### Whole-Genome Sequencing

Prior to WGS, a primary species identification from single colonies was performed by matrix-assisted laser desorption ionization time-of-flight mass spectrometry (MALDI-TOF MS) (Bruker, Billerica, MA, United States) and MBT Compass IVD software 4.1.60 (Bruker) as described by Halbedel et al. (2020).

As for WGS, DNA was isolated from bacterial cultures with the MagAttract HMW DNA Kit (Qiagen, Hilden, Germany) according to the manufacturer’s instructions for Gram-positive bacteria. The amount of input DNA was quantified on a Lunatic instrument (Unchained Labs, Pleasanton, CA, United States). Nextera XT chemistry (Illumina Inc., San Diego, CA, United States) was used to prepare sequencing libraries for a 300 bp paired-end sequencing run on an Illumina MiSeq sequencer. Samples were sequenced to achieve a minimum 80-fold coverage using recommended standard protocols by Illumina. The resulting FASTQ files were quality trimmed and *de novo* assembled with the SPAdes version 3.9.0. Contigs were filtered for a minimum of fivefold coverage and 200 bp minimum length with SeqSphere+ software v. 7.8.0 (Ridom, Münster, Germany) (Jünemann et al., 2013).

### Serotype, Sequence Type, and Core Genome Multilocus Sequence Typing of *Listeria monocytogenes*

From the WGS of the *L. monocytogenes* strains, serotypes were determined by the sequence-specific extraction of targets using the *L. monocytogenes* 5-plex PCR Serogroup task templates of the SeqSphere+ v. 7.8.0 (2021-7) software with fragments from five DNA regions (*lmo118, lmo0737, ORF2110, ORF2829*, and *prs* as an internal amplification control) previously described by Doumith et al. (2004) and Lee et al. (2012).

The sequence type (ST) was determined with Task templates for available MLST schemes from the SeqSphere+ v. 7.8.0 (2021-7) software (Jünemann et al., 2013). The ST was confirmed in the strains with fragments from the seven housekeeping genes *abcZ, bglA, cat, dapE, dat, Idh*, and *ihkA* (Ruppitsch et al., 2015a; Moura et al., 2016) and with the profiles from the Institut Pasteur MLST *Listeria* database^1^.

The cgMLST was performed on the basis of the profile of 1,701 loci of cgMLST complex types (CTs) (Ruppitsch et al., 2015a) with Task templates for SeqSphere+ v. 7.8.0 (2021-7). We defined a cgMLST cluster as the group of isolates with less than10 different alleles among the studied strains. We used SeqSphere in the mode that ignored pairwise missing values and an unweighted pair group method with arithmetic mean to generate phylogenetic trees (Halbedel et al., 2020).

### Antibiotic Resistance Profile

The disk diffusion method was applied based on the recommendations of the Clinical and Laboratory Standards Institute (Clinical and Laboratory Standards Institute [CLSI], 2018). The commercial antibiotic disks included 10 μg ampicillin (AMP), 10 μg penicillin (PEN), 25 μg sulfamethoxazole-trimethoprim (STX), 15 μg erythromycin (ERY), 30 μg vancomycin (VAN), tetracycline (TET) 30 μg, ciprofloxacin (CIP) 5 μg, and 30 μg chloramphenicol (CHL). The resistance/susceptibility profiles of the strains were characterized by measuring the zone of inhibition and interpreting the inhibition diameters according to the manufacturer’s instructions; *Streptococcus pneumoniae* ATCC 49619 was used as a reference. In addition, *E. coli* ATCC 25922 and *L. monocytogenes* ATCC 7644 were used as controls.

### Virulence Genes Amplification

The method described by Aznar and Alarcón (2002) was used to amplify conserved regions of the three characteristic virulence genes listeriolysin O (*hlyA*) (Border et al., 1990), positive regulatory factor A (*prfA*) (Klein and Juneja, 1997), and internalin A (*inlA*) (Montero et al., 2015). The genomic DNA of the suspected strains was extracted and purified with the UltraClean Microbial DNA Isolation Kit (Mo Bio Laboratories, Qiagen, Carlsbad, CA, United States) and mixed with GoTaq Green Master Mix (Promega, Madison, WI, United States) in a thermocycler (Fermelo Biotec, China). Using an agarose gel imaging system, the amplified products were stained and visualized on 1.5% agarose gel with a 1.0 mg/mL ethidium bromide solution.

### *In silico* Detection of Virulence and Antibiotic Resistance Genes

Virulence genes were established with the task template VFDB 2.0 feature in SeqSphere+ for WGS data (Chen et al., 2016). Thresholds were set for the target scanning procedure as a required identity ≥ 90% with the reference sequence and an aligned reference sequence ≥ 99%. The Comprehensive Antibiotic Resistance Database (CARD) was used with the default “perfect” and “strict” settings for the sequence analysis of antimicrobial resistance genes (Jia et al., 2017). The Task Template AMRFinderPlus 3.2.3 available in the Ridom SeqSphere+ 7.8.0 software was used with the EXACT method at the 100% setting together with the BLAST alignment of protein sequences against the AMRFinderPlus database (Feldgarden et al., 2019).

### *In silico* Detection of Plasmids and Mobile Genetic Elements

The PlasmidFinder 2.1 and MobileElementFinder 1.0 tools were used to detect plasmids and MGEs. The selected minimum identity was 95 and 90%, respectively^2^ (Carattoli et al., 2014; Johansson et al., 2021).

### Bioinformatic Search of CRISPR-Cas Loci

The search for and characterization of CRISPR arrays and their association with Cas proteins was determined with CRISPRCasFinder and CRISPRminer (Couvin et al., 2018; Zhang et al., 2018), which are available at https://crisprcas.i2bc.paris-saclay.fr and http://www.microbiome-bigdata.com/CRISPRminer. The following parameters were used: 18–55 bp repeated sequence length, 25–60 bp spacer length, 0.6–2.5 spacer sequence size as a function of repeated sequence size, and 60% maximum percentage similarity between spacers. Phages associated with sequence spacers were also determined with the CRISPRminer program (Zhang et al., 2018).

The CRISPR systems were determined with the CRISPRmap program (Lange et al., 2013). The CRISPRTarget program was used to determine the protospacer adjacent motif (PAM) sequences associated with each repeated sequence of the identified arrays.

## Results

### Prevalence of *Listeria monocytogenes*

In total 3.1% (14/436) of samples were positive for *L. monocytogenes* when using the VIDAS system. In terms of food group, the highest positivity, 36% (8/22), occurred in pre-processed fruits and vegetables, followed by 5.9% (4/67) for prepared meals and dishes, 1.1% (2/186) for cooked meats, and 0% (0/161) for cheese and fresh cheese (Table 1).

**TABLE 1:** Positivity of *Listeria monocytogenes* in risk food groups.

Foods groups	*n*	Positives	%
Cheeses	161	0	0
Cooked meats	186	2	0,4
Pre-processed fruits and vegetables	22	8	1,8
Meals and mixed dishes with raw and/or cooked ingredients	67	4	0,9
Total	436	14	**3,1**

Fourteen strains were isolated from the positive samples and confirmed as *L. monocytogenes* by MALDI-TOF MS.

### Core Genome Multilocus Sequence Typing, Sequence Type, Complex Type, Clonal Complex, and Serotype From Whole-Genome Sequencing of *Listeria monocytogenes*

**FIGURE 1:**
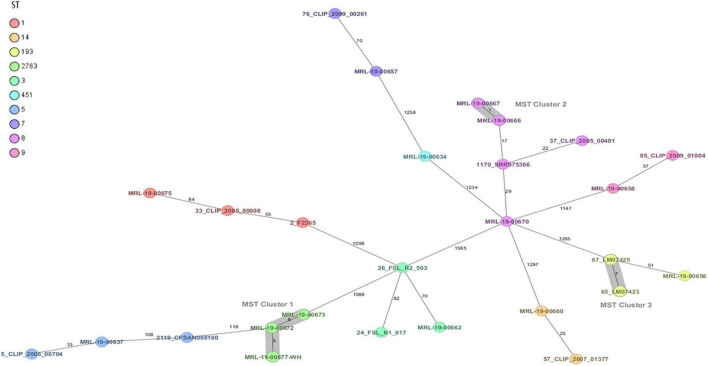
Minimum spanning tree (MST) of 14 *Listeria monocytogenes* strains from ready-to-eat foods isolated in Chile. *L. monocytogenes* strains with ST1, ST3, ST5, ST7, ST8, ST9, ST14, ST193, ST451, and ST2763 are of clinical and food origin. Calculation of the MST is based on the defined core genome multilocus sequence typing (cgMLST) scheme consisting of 1,701 target genes from Task templates for SeqSphere+ v. 7.8.0 (2021-7). Isolates are represented as colored circles according to the classical MLST. Black numbers are in accordance with the allelic difference between isolates. Isolates with closely related genotypes are marked as Cluster.

Whole-genome sequencing using cgMLST grouped the strains into two clusters and nine unrelated complex types (CTs) (Figure 1). In addition, the 14 strains were grouped in 10 STs and 9 CCs using average nucleotide identity (ANI), ribosomal MLST, and cgMLST complex type (CT).

The dominant serotype was 1/2a in seven strains, of which three strains were identified as ST8 (CC8, CT8068, and CT8004), one ST7 (CC7 and CT8064), one ST14 (CC14 and CT8065), one ST193 (CC193 and CT8063), and one ST 451 (CC11 and CT4117).

Furthermore, the serotype 1/2b was found in five strains, one ST3 (CC3 and CT8066), one ST5 (CC5 and CT8052), and three new STs. One strain showed serotype 4b (ST1, CC1, and CT 8007), and one strain was serotype 1/2c (ST9 (CC9 and CT5231) (Table 2). A new ST was identified as ST2763 (CC5 and CT8006) in the three strains, and was uploaded to the Institute Pasteur MLST Listeria database.

**TABLE 2:** Identification of *L. monocytogenes* strains by matrix-assisted laser desorption ionization time-of-flight mass spectrometry (MALDI-TOF MS) and whole-genome sequencing (WGS).

Sample ID	Food	MALDI-TOF MS	WGS	ST	CC	CT	Serotype
MRL-19-00634	Strawberries	*Listeria monocytogenes*	*Listeria monocytogenes*	451	11	4117	1/2a
MRL-19-00637	Cooked shrimp	*Listeria monocytogenes*	*Listeria monocytogenes*	5	5	8052	1/2b
MRL-19-00656	Cooked sausage	*Listeria monocytogenes*	*Listeria monocytogenes*	193	193	8063	1/2a
MRL-19-00657	Strawberries	*Listeria monocytogenes*	*Listeria monocytogenes*	7	7	8064	1/2a
MRL-19-00658	Grapes1/2a	*Listeria monocytogenes*	*Listeria monocytogenes*	9	9	5231	1/2c
MRL-19-00660	Coleslaw	*Listeria monocytogenes*	*Listeria monocytogenes*	14	14	8065	1/2b
MRL-19-00662	Mix salads	*Listeria monocytogenes*	*Listeria monocytogenes*	3	3	8066	1/2b
MRL-19-00666	Mushrooms	*Listeria monocytogenes*	*Listeria monocytogenes*	8	8	8068	1/2a
MRL-19-00667	Mushrooms	*Listeria monocytogenes*	*Listeria monocytogenes*	8	8	8068	1/2a
MRL-19-00670	Pâté	*Listeria monocytogenes*	*Listeria monocytogenes*	8	8	8004	1/2a
MRL-19-00672	Fettuccine	*Listeria monocytogenes*	*Listeria monocytogenes*	2763	5	8006	1/2b
MRL-19-00673	German roast	*Listeria monocytogenes*	*Listeria monocytogenes*	2763	5	8006	1/2b
MRL-19-00675	Spinach salad	*Listeria monocytogenes*	*Listeria monocytogenes*	1	1	8007	4b
MRL-19-00677	Pot roast	*Listeria monocytogenes*	*Listeria monocytogenes*	2763	5	8006	1/2b

*ST, sequence type; CC, clonal complex; CT, complex type.*

### Antibiotic Resistance Profile and *in vitro* Detection of Virulence Genes

Most of the strains 78% (11/14) were susceptible to all the antibiotics; only the MRL-19-00656, MRL-19-006573, and MRL-19-00662 strains were resistant to ampicillin.

Regarding the virulence genes, all strains in the present study amplified *hlyA, prfA*, and *inlA* genes (Table 3).

**TABLE 3:** Detection of putative virulence genes and antibiotic resistance profile of *L. monocytogenes* strains.

Strain	Source	Antibiotics									Genes		
		ST	AMP	PEN	STX	ERY	VAN	TET	CIP	CHL	*hlyA*	*prfA*	*inlA*
MRL-19-00634	Strawberries	451	S	S	S	S	S	S	S	S	+	+	+
MRL-19-00637	Cooked shrimp	5	S	S	S	S	S	S	S	S	+	+	+
MRL-19-00656	Cooked sausage	193	R	S	S	S	S	S	S	S	+	+	+
MRL-19-00657	Strawberries	7	R	S	S	S	S	S	S	S	+	+	+
MRL-19-00658	Grapes	9	S	S	S	S	S	S	S	S	+	+	+
MRL-19-00660	Coleslaw	14	S	S	S	S	S	S	S	S	+	+	+
MRL-19-00662	Combination salads	3	R	S	S	S	S	S	S	S	+	+	+
MRL-19-00666	Mushrooms	8	S	S	S	S	S	S	S	S	+	+	+
MRL-19-00667	Mushrooms	8	S	S	S	S	S	S	S	S	+	+	+
MRL-19-00670	Pâté	8	S	S	S	S	S	S	S	S	+	+	+
MRL-19-00672	Fettuccine	2763	S	S	S	S	S	S	S	S	+	+	+
MRL-19-00673	German roast	2763	S	S	S	S	S	S	S	S	+	+	+
MRL-19-00675	Spinach salad	1	S	S	S	S	S	S	S	S	+	+	+
MRL-19-00677	Pot roast	2763	S	S	S	S	S	S	S	S	+	+	+

*R, resistance; S, susceptibility; Genes: hlyA, listeriolysin O; prfA, positive regulatory factor A; inlA, internalin A.*

### *In silico* Detection of Virulence and Antibiotic Resistance Genes

**FIGURE 2:**
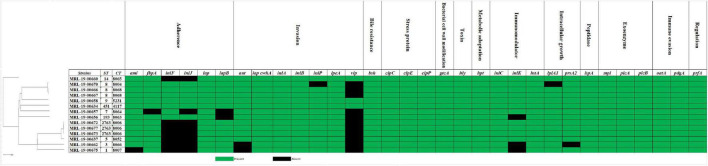
Distribution of virulence genes present in 14 *L. monocytogenes* strains isolated from ready-to-eat foods. Green boxes indicate the presence of the gene and black boxes its absence.

The virulence factor database (VFDB) was used to evaluate the 33 major virulence gene. All the strains had the following genes: *bsh* (bile resistance), *clpCEP* (stress protein), *hly* (toxin-listeriolysin O precursor), *hpt* (metabolic adaptation), *iap/cwhA*, *inlA*, *inlB*, and *ipeA* (invasion), *lspA* (peptidase), *mpl*, *plcA*, *plcB* (exoenzyme), *oat*, *pdgA* (immune evasion), and *prfA* (regulation) (Figure 2). Only the MRL-19-00675 genome strain exhibited the *Listeria* pathogenicity island 3 (LIPI-3). A mutation in position 2054 (G:A) of *inlA* gene was found in the MRL-19-00658 strain, known as premature stop codons (PMSC) type 11. A new mutation in position 819 of the *inlA* gene was encountered in the MRL-19-00662 strain, which was not identified because this mutation (deletion of A) has not yet been described in the literature or in the *inlA* PMSC profiles of the Institut Pasteur MLST *Listeria* database (see Text Footnote 1).

Furthermore, genes associated with biofilm formation such as *cheY*, *inlL*, *prfA*, *actA*, *lmo0673*, and *lmo2504* were identified in all the strains; these genes play an important role in the survival and persistence of *L. monocytogenes*. The *bapL*, *recO*, and *luxS* genes were not found in any strain. The antimicrobial *fosX*, *lin*, *norB*, and *mprF* resistance genes were identified in all *L. monocytogenes* strains. These genes confer resistance to fosfomycin, lincosamides, quinolones, and cationic peptides that disrupt the cell membrane such as defensins. Regarding the genes that confer resistance to tetracycline, the *tetA* and *tetC* genes were detected and *tetM* and *tetS* were absent in all the strains. In addition, the *arsBC* and *bcrBC* genes were identified in all strains, which confer resistance to stress, and the *clpL* gene, which confers resistance to disinfectants.

### Detection of Plasmids and Mobile Genetic Elements

Plasmids were found in 85% (12/14) of the strains. Inc18(rep25) was detected in eight strains, Inc18(rep26) in three, and N1011A in one. In addition, the ST2763 harbored the Inc18(rep25) and rep3(rep32) plasmids (Table 4).

**TABLE 4:** Plasmids identified in *Listeria monocytogenes* strains by plasmidFinder tool.

Strains	Plasmids	Identity (%)	Length	Note	Accession number	Function
MRL-19-00637	Inc18(rep26)	100	1,650	M643p00680(N1011Aplasmid)	CP006611	Antibiotic resistance
MRL-19-00656	Inc18(rep25)	99.7	1,761	pLM330006(pLM33)	GU244485	
MRL-19-00658	Inc18(rep25)	99.8	1,761	pLM330006(pLM33)	GU244485	
MRL-19-00662	Inc18(rep25)	100	1,761	pLM330006(pLM33)	GU244485	
MRL-19-00666	Inc18(rep26)	98.8	1,809	repA(pLGUG1)	FR667693	
MRL-19-00666	Inc18(rep26)	98.8	1,809	repA(pLGUG1)	FR667693	
MRL-19-00670	Inc18(rep26)	98.8	1,809	repA(pLGUG1)	FR667693	
MRL-19-00672	Rep3(rep32)	99,9	1,161	pli0023(pLI100)	AL592102	
	Inc18(rep25)	100	1,761	pLM330006(pLM33)	GU244485	
MRL-19-00673	Inc18(rep25)	100	1,761	pLM330006(pLM33)	GU244485	
	Rep3(rep32)	99.9	1,161	pli0023(pLI100)	AL592102	
MRL-19-00675	Inc18(rep25)	100	1,767	M640p00130(J1776plasmid)	CP006612	
MRL-19-00677	Rep3(rep32)	100	1,161	pli0023(pLI100)	AL592102	
	Inc18(rep25)	100	1,761	pLM330006(pLM33)	GU244485	

The MGEs (insertion sequences, transposons) were found in only nine strains. The most frequent MGEs were ISLmo3, ISLmo5, ISLmo7, ISLmo9, ISLmo8, ISS1N, cn_8625_ISS1N, CN_12410_ISS1N, and CN_8566_ISS1N (Supplementary Table 1).

### CRISPR-Cas Loci

Genome analysis showed the presence of CRISPR-Cas systems in 71% (10/14) of the genomes. These systems consist of at least one array; however, between two and five arrays can be observed in 50% (5/10) of the genomes in different positions. The arrays had among 3 repeated sequences and 2 spacers and up to 28 repeated sequences and 27 spacers (Table 5).

**TABLE 5:** CRISPR-Cas systems identified in *L. monocytogenes* genomes.

Strains	Operon structure type	Position	Maximum number of spacers per strains	Number of CRISPRs arrays per strain	Repeat consensus	Cas genes
MRL-19-00634	CAS-Type II-B	63376–63983 45173–46922	9 27	10 28	ATTTACATTTCATAATAAGTAGTTAAAAC ATTACTTACATCAACTTCTTCAAGGCTAGTACAA	*cas3, cas2, cas1, cas4, cas3, cas5, cas7, cas8b2, cas6, csa3*
MRL-19-00656	CAS-Type II-B	35097–35359	4	5	ATTTACATTTCACAATAAGTAACTAAAACAT	*cas3, DinG, cas3, casR, csa3*
MRL-19-00657	CAS-Type II-B	25–157 11508–11772 13778–14229 25–1743 422853–422592	2 4 7 26 4	3 5 8 27 5	GTTTTGGTAGCATTCAAAATAACATAGCTCTAAAAC GTTTTGGTAGCATTCAAAATAACATAGCTCTAAAAC ATTTACATTTCACAATAAGTAACTAAAAC GTTTTGGTAGCATTCAAAATAACATAGCTCTAAAAC ATTTACATTTCACAATAAGTAACTAAAAC	*cas9, cas1, cas2, csn2, cas3, DinG*
MRL-19-00658	CAS-Type II-B	161775–162035	4	5	ATTTACATTTCACAATAAGTAACTAAAAC	*cas2*
MRL-19-00660	CAS-Type II-B	7873–8259 7168–8658 45319–45643	6 23 5	7 24 6	ATTTACATTTCAAAATAAGTAACTAAAAC ATTTACATTTCAAAATAAGTAACTAAAAC ATTTACATTTCAAAATAAGTAACTAAAAC	*cas6, csa3, cas8b2, cas7, cas5, cas3, cas1, cas2, csa3*
MRL-19-00662	CAS-Type II-B	15–400 6877–8133 38278–29474	6 16 3	7 17 4	ATTTACATTTCATAATAAGTAGTTAAAAC GTTTTAGAGCTATGTTATTTTGAATGCTACCAAAAC ATTTACATTTCATAATAAGTAGTTAAAAC	*csn2, csa3, cas3, cas2, cas1, cas9, cas5, cas7, cas8b2, csa3, cas6, casR, DinG, DEDDh, cas3, cas5*
MRL-19-00666	CAS-Type II-B	268974–269231	4	5	ATTTACATTTCACAATAAGTAACTAAAAC	*cas3*
MRL-19-00667	CAS-Type II-B	12064–12645	9	10	ATTTACATTTCACAATAAGTAACTAAAAC	*cas3, casR*
MRL-19-00670	CAS-Type II-B	7178–7434	4	5	ATTTACATTTCACAATAAGTAACTAAAAC	*cas3, casR, DEDDh, DinG, csa3*
MRL-19-00675	CAS-Type II-B	19–161 62–1072	2 15	3 16	ATTTCAATCCTCTAACTCTAAACAGAGTTAGTC TGTTAAACTTCCAAAGGTAACCTCTATTGGTAATG CTACATTT	*cas3, cas5, DinG, DEDDh, csa3, cas1, cas4, cas8c, cas7, cas8c*

*The characteristic repeated sequences of the identified CRISPR arrays and their position in the genome are shown.*

**FIGURE 3:**
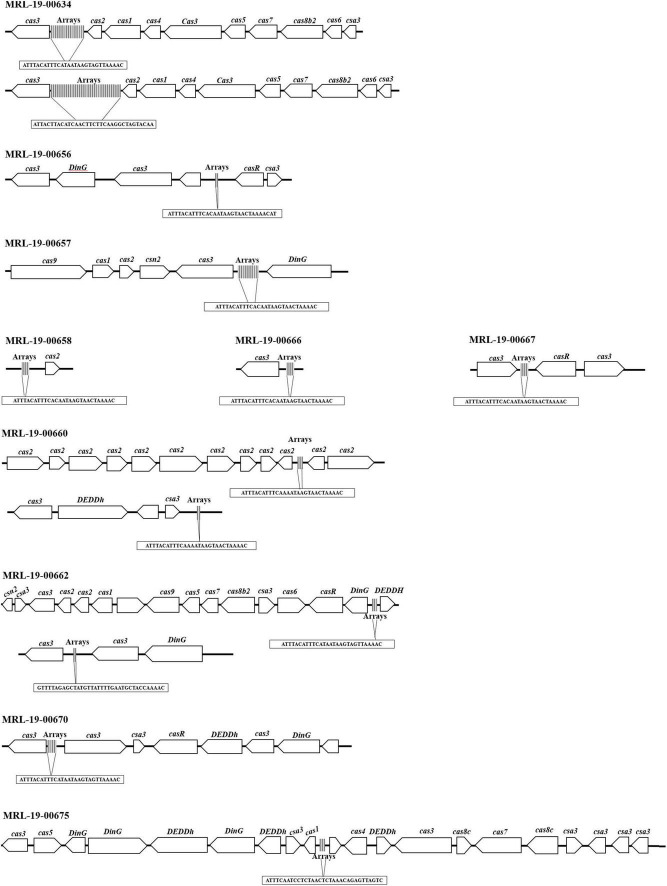
CRISPR-Cas systems identified in *L. monocytogenes* genomes. The identified systems belong to the CRISPR-Cas type II-B system, and some genomes show more than one array.

Using the CRISPRmap program, the repeated sequences and the associated cas genes enabled to determine the identified CRISPR systems to type II-B (Figure 3 and Table 1). In two of the arrays, only one *cas*-associated gene was identified; in contrast, the rest of the genomes showed sequences that encoded up to 16 proteins associated with these CRISPR-Cas systems.

The analysis of the spacer and PAMs enabled us to associate them to sequences corresponding to different bacteriophages, which are associated with the *Listeria* genus (Supplementary Tables 2, 3).

The bioinformatics analysis of the genomes enabled the detection of protein sequences associated with the AcrIIA1 and AcrIIA3 Anti-CRISPR systems in the three studied genomes MRL-19-00657, MRL-19-00658, and MRL-19-00660 (Table 6), which are associated with phages present in these genomes.

**TABLE 6:** Anti-CRISPR elements.

Bacteria ID	Anti-CRISPR message	*E*-value	Match range	Coverage	Protein sequence
MRL-19-00657_contig8_ 134202_134651_+	gb| AEO04364.1| gp28 [Listeria monocytogenes J0161]	1.75E-107	0.986577181	1	MTIKLLDEFLKKHDLTRYQLSKLTGISQNTLKDQNEKPLNK YTVSILRSLSLISGLSVSDVLFELEDIEKNSDDLAGFKHLLD KYKLSFPAQEFELYCLIKEFESANIEVLPFTFNRFENEEHVN IEKDVCKALENAITVLKEKKNELI
MRL-19-00658_contig1_ 1720_2097_−	emb| CBY03209.1| bacteriophage protein GP30 [Listeria monocytogenes serotype 7 str. SLCC2482]	3.42965e-75	0.864	1	MYNKAEIMKQAWNWFTDSNVWLSDIEWVSYTDKEKTFS VCLKAAWSKAKEEVKEVEKEIKHISKSEELKAWNWAERK LGLRFNISDDEKFTSVKDETKQHFGLSVWACAMKAVKLH NDLFPQTAA
MRL-19-00658_contig1_ 884_1333_−	gb| AEO04364.1| gp28 [Listeria monocytogenes J0161]	4.35583e-91	0.751677852349	1	MSIKLLDEFLKKHSKTRYQLSKLTGISQNTLNDYNKKELNK YSVSFLRALSMCAGISTFDVFIELAELEKSYDDLAGFKHLL DKYKLSFPAQEFELYCLIKEFESANIEVLPFTFNRFENEEHV NIEKDVCKALENAITVLKEKKNELL
MRL-19-00660_contig12_ 68115_68564_+	gb| AEO04364.1| gp28 [Listeria monocytogenes J0161]	1.70745e-108	1.0	1	MTIKLLDEFLKKHDLTRYQLSKLTGISQNTLKDQNEKPLNK YTVSILRSLSLISGLSVSDVLFELEDIEKNSDDLAGFKHLLD KYKLSFPAQEFELYCLIKEFESANIEVLPFTFNRFENEEHVNI KKDVCKALENAITVLKEKKNELL
MRL-19-00660_contig31_ 32482_32859_+	emb| CBY03209.1| bacteriophage protein GP30 [Listeria monocytogenes serotype 7 str. SLCC2482]	4.3182e-75	0.904	1	MYNKSEIMQQAWNWFRDSSVWLSDIEWVSYTDKEKTFSV CLKAAWSKAKEEVEESKKESKHIAKSEELKAWNWAESKL GLRFNISDDEKFTSVKDETKINFGLSVWACAMKAVKLHN DLFPQTAA
MRL-19-00660_contig31_ 33246_33695_+	gb| AEO04364.1| gp28 [Listeria monocytogenes J0161]	2.02671e-86	0.697986577181	1	MSIKLLDEFLKKHSKTRYQLSKLTGISQNTLNDYNKKELN KYSVSFLRALSMCAGISTFDVFIELAELEKSYDDLAGFKHL LDKYKLSFPAQEFELYCLIKEFECANIEVLPFTFNRFENETH VDIEKDVRKALENAITVLKEKKNELI

*Anti-CRISPR protein sequences identified in the studied genomes are shown.*

## Discussion

*Listeria monocytogenes* persists as a relevant public health and food safety risk due to its ubiquity, persistence under adverse environmental conditions, and pathogenicity (Hurley et al., 2019; Chen et al., 2020a).

In the present study, general positivity for *L. monocytogenes* in RTE foods was 3.1% (14/436). Positivity for *L. monocytogenes* in RTE foods in different countries has been reported as 5.5% in China (Li et al., 2018), 7.5% in Chile (Bustamante et al., 2020), 11.9% in Uruguay (Braga et al., 2017), 8.5% in Turkey (Sanlibaba et al., 2018), and 13.5% in Poland (Szymczak et al., 2020). Bustamante et al. (2020) reported prevalence values of 17.5, 8.6, and 8.5% in prepared meals and dishes, pre-processed fruits and vegetables, and cooked meats, respectively. Furthermore, positivity for *L. monocytogenes* was 0% in dairy products and cheeses, which concurs with the present study, and this situation is noteworthy because dairy products and cheeses have been associated in recent years with many outbreaks in Europe and the United States (Fretz et al., 2010; Amato et al., 2017; Martínez-Rios and Dalgaard, 2018; Churchill et al., 2019). Therefore, a better understanding of the ecology and biology of *L. monocytogenes* that focuses on virulence factors and stress response would further improve the control of this important foodborne pathogen (Ryser, 2021).

The *L. monocytogenes* strains in the present study revealed that ST8 was the most prevalent ST from samples of RTE vegetables and pork pâté. The ST8 has been found in different RTE foods such as meats, salmon, cooked meats, fried rice and noodles, and vegetables (Wang et al., 2015; Ziegler et al., 2018; Chen et al., 2020b). In addition, ST8 has been responsible for cases of human listeriosis in Canada, Italy, Switzerland, and Germany (Knabel et al., 2012; Mammina et al., 2013; Althaus et al., 2014; Ruppitsch et al., 2015b; Halbedel et al., 2020), and it has been considered to have high pathogenic potential (Fagerlund et al., 2016). Different authors state that *L. monocytogenes* ST 8 is one of the most persistent STs in RTE food processing plants; hence, there is a permanent risk of food recontamination by this pathogen (Knudsen et al., 2017). The second most prevalent was ST2763 (CC5), which is a new ST found in the present study and isolated in meats and RTE prepared dishes. There was a diverse distribution of the other STs, including ST1, ST3, ST5, ST7, ST9, ST14, ST193, and ST451, which have been isolated in outbreaks, clinical cases, and different foods (Althaus et al., 2014; Amajoud et al., 2018; Cabal et al., 2019; Ulloa et al., 2019; Halbedel et al., 2020). Six of the fourteen *L. monocytogenes* strains belonged to serogroup IIa (serotype 1/2a; ST7, ST8, ST193, and ST451), six to serogroup IIb (serotype 1/2b; ST3, ST5, ST14, and ST2763), one to serogroup IVb (serotype 4b; ST1), and one to serogroup IIc (serotype 1/2c; ST9). These four serotypes have been associated with more than 98% of reported cases of listeriosis worldwide (Gorski, 2021).

The treatment for listeriosis includes antibiotics such as ampicillin, tetracyclines, amoxicillin, and sulfamethoxazole (Thønnings et al., 2016). In the present study, 11 isolates were susceptible to all the antibiotics, while only 3 exhibited resistance to ampicillin. This is a cause for concern because previous reports in Chile have indicated the susceptibility of *L. monocytogenes* to ampicillin and also because ampicillin and amoxicillin are currently used to treat this infection (Seoane, 2013; Kumaraswamy et al., 2018). Several authors have encountered resistance to ampicillin in *L. monocytogenes* strains isolated from raw and cooked meats and fish products with a prevalence between 6 and 83% (Yucel et al., 2005; Jamali et al., 2013; Arslan and Baytur, 2019; Bustamante et al., 2020; Maćkiw et al., 2020). Emerging resistance to penicillin in clinical strains poses a major public health concern because penicillin is the standard treatment for human listeriosis (Martínez et al., 2001). Therefore, the fact that we detected ampicillin-resistant *L. monocytogenes* strains in the present study should alert authorities and food manufacturers to the latent risk associated with the consumption of these RTE foods contaminated by this pathogen.

We found the presence of resistance genes with mechanisms of antibiotic efflux (*norB*), antibiotic target alteration (*mprF*), and antibiotic inactivation (*lin, fosX*). In addition, our study reported genes that confer resistance to tetracycline (*tetA* and *tetC*). Wilson et al. (2018) reported that all strains displayed the resistance gene to fosfomycin (*fosX*); however, they did not detect any genes associated with tetracycline (*tetA*) or erythromycin (*ermABC*). This differs from our study in which the *fosX* and *tetA* genes were identified. Mafuna et al. (2021) encountered resistance genes in strains such as *fosX*, *lin*, *mprF*, and *norB*, and they reported an increasing global trend of resistance genes present in the food chain. *L. monocytogenes* is currently considered to be intrinsically resistant to fosfomycin because of the lack of expression in the membrane transport systems and a natural resistance to lincomycin due to the ribosomal protection of an ATP-binding cassette F (ABC-F) protein (Mota et al., 2020).

However, there was a difference in our study between the prediction of resistance genes and antibiotic susceptibility testing, which is due to the existence of intrinsic resistance according to some authors (Cox and Wright, 2013); in addition, the resistance genes are ancient and predate the use of antibiotics (Kashuba et al., 2017; Peterson and Kaur, 2018). Gygli et al. (2019) reported that the possible discrepancy between gene detection by WGS and antibiotic susceptibility testing of *Mycobacterium tuberculosis* strains could arise because the clinical concentrations established to classify it as resistant have cutoff scores that are too high, thus misclassifying strains as susceptible. Aljahdali et al. (2020), found a positive concordance between the presence of resistance genes and resistance phenotypes in various in *Salmonella* strains; however, they also observed that some strains with beta-lactamase resistance genes were still phenotypically susceptible to amoxicillin-clavulanic acid. Therefore, the difference between genotype and phenotype in these strains could be due to exceptional mutations that reduce gene expression and can confer susceptibility to antimicrobial agents used in susceptibility tests. With the discovery of varied antimicrobial resistance genes and gene transfer mechanisms, non-genetic mechanisms mediated by small molecules can alter the phenotypic susceptibility to antibiotics of bacterial cells (El-Halfawy and Valvano, 2012).

The presence of resistance genes to quaternary ammonium, stress, and biofilm formation have been described as key factors for the adaptation and survival in food processing plants (Horlbog et al., 2018). We identified the *bcrBC* cassette in three ST2763 strains and one ST5 strain, which were all serotype 1/2b associated with persistence and resistance to benzalkonium chloride, a common disinfectant used in the food industry (Cooper et al., 2021). In addition, the *clpL* gene was detected in one ST3 strain and one ST9 strain both isolated from RTE vegetables. This gene has been identified as an important predictor of heat resistance of *L. monocytogenes* (Pöntinen et al., 2017).

All our strains amplified the three evaluated virulence factors *in vitro* and confirmed by detection of these genes *in silico*. Among these strains, the most studied virulence genes are *hlyA, prfA*, and *inlA*. The *hlyA* gene encodes listeriolysin O, which allows pore formation for pathogen entry into the cells; it is only present in virulent species of *Listeria* spp. and is widely used to assess the presence of the virulence factor in *L. monocytogenes* isolated in RTE foods (Churchill et al., 2006; Abdollahzadeh et al., 2016). The PrfA protein is indispensable for virulence gene expression (including the *prfA* gene) in pathogenic species of *L. monocytogenes*, and it depends on environmental conditions such as high temperature and stress (Aballa et al., 2019). Internalin A (*inlA*) was found in all the studied isolates and is considered relevant because it participates in the adherence process between the bacteria wall and the intestinal cells (Drolia and Bhunia, 2019). Recent studies have focused on the presence of premature stop codons (PMSC) in the *inlA* gene, which results in impaired virulence; this type of mutation is more frequent in food isolates than in clinical cases (Van Stelten et al., 2010; Ferreira da Silva et al., 2017). Only one isolate in our study showed a PMSC with a type II mutation (G:A) in position 2054, generating a truncated internalin of 684 amino acids as describe recently (Van Stelten and Nightingale, 2008). However, we detected a new mutation in the MRL-19-00662 strain (ST3, CC3, and serotype 1/2b) in position 819 (deletion of A), resulting in a truncated internalin of 273 amino acids, which has not yet been reported in the literature. Nine strains in our study have pathogenic potential; it was confirmed that the prevalent STs, such as ST1 (CC1) and ST9 (CC9), are associated with listeriosis outbreaks in Chile and have persisted over time (Cantinelli et al., 2013; Montero et al., 2015; Toledo et al., 2018; Cabal et al., 2019; Ulloa et al., 2019).

A characteristic trait of *Listeria* plasmids is the presence of many MGEs encoding transposases, such as insertion sequences (IS) and transposons, and other recombinases that are determinants in the dissemination of adaptive foreign DNAs and resistance (Kuenne et al., 2010). The most common plasmids were inc18(rep25), inc18(rep26), and rep3(rep26), and only one strain showed N1-011A. The plasmid incompatibility group inc18 is naturally found in *Streptococcus* and *Enterococcus* spp. (Zhu et al., 2010) and encodes a variety of resistance to antibiotics due to their overuse in environmental and food settings (Kohler et al., 2018). In addition, plasmid N1-11A has been found in RTE seafood processing plants in France and in the food chain in South Africa: it is associated with the resistance to disinfectants such as benzalkonium chloride (Mafuna et al., 2021).

CRISPR-Cas systems are acquired immunity systems that allow bacteria and archaea to acquire exogenous material from bacteriophages and plasmids (Hupfeld et al., 2018). The CRISPR-Cas systems is a possible involved in the regulation of gene expression, including virulence genes, which have been described in a number of pathogens (Louwen et al., 2014). It was possible to determine that the repeated sequences and associated *cas* genes in the studied *L. monocytogenes* strains corresponded to type II-B systems and that the presence of the *cas8b* and *cas9* genes allowed their classification in subtype B. However, the arrays could be related to one cas gene, likewise in the systems that only show sequences that encode for *cas3* and *cas2*. Kuenne et al. (2013) studied CRISPR-Cas in three different loci of *L. monocytogenes* strains. CRISPR-Cas locus 1 was characterized by a single CRISPR matrix, locus 2 belonged to type I-B, and locus 3 was classified as type II-A. CRISPR-Cas locus 1 was previously found as being associated with the presence of a tracrRNA, which is suggested to control virulence in *L. monocytogenes* strain 1/2a EGD-e during growth in macrophages; however, it is still unknown how this track RNA could control virulence (Mraheil et al., 2011). Louwen et al. (2013) showed that the ability to translocate through intestinal walls was suppressed when deleting cas9 in *Campylobacter jejuni* isolates, which affected virulence. The same authors reported that supplementing *C. jejuni* isolates with cas9, which does not have a CRISPR-Cas system, significantly increased virulence in this pathogen This can also be associated with the array size because those in which these genes are absent have smaller arrays.

As for the spacer sequences, they provide us with the history of the invasive elements to which the bacterium has been subjected because these sequences are associated with exogenous material. For arrays identified in the present study, spacers were related to sequences corresponding to bacteriophages that specifically infected the *Listeria* genus. Therefore, those bacteria that have this information are able to evade infection by these bacteriophages, unlike those that do not. The phages have also been able to develop strategies in response to CRISPR-Cas, such as the Anti-CRISPR proteins, which were identified in the genomes under study. It has been determined for *L. monocytogenes* that the prophages show anti-Cas9 proteins such as AcrIIA1, which successfully blocks and inactivates Cas9 (Osuna et al., 2020). These proteins were identified in 36% of the genomes in the present study that showed CRISPR-Cas systems. Even though these strains show systems that allow the acquisition of exogenous material and the possibility of evading infection by bacteriophages, the presence of these phages with Anti-CRISPR proteins evade these immunity mechanisms acquired by the bacteria and thus counteract the acquired immunity (Hynes et al., 2018).

## Conclusion

*Listeria monocytogenes* strains isolated from RTE foods exhibited multiple virulence factors and antibiotic resistance factors after *in vitro* and *in silico* analyses. It is therefore necessary to perform continuous genomic surveillance on these foods because of the risk associated with *L. monocytogenes* contamination and their consumption by populations at risk.

## Data Availability Statement

The datasets presented in this study can be found in online repositories. The names of the repository/repositories and accession number(s) can be found in the article/Supplementary Material.

## Author Contributions

JP-F, OH, FB, SL, AP, AC-F, AC-C, MT, GF, and WR conceived the experiments and prepared the manuscript. JP-F, FB, AC-F, JM-R, CC, CO, JX-C, MA-L, SL, and MT conducted the laboratory work. JP-F, OH, AC-C, JX-C, JM-R, SL, and WR drafted the manuscript. All authors reviewed and approved the final manuscript.

## Conflict of Interest

The authors declare that the research was conducted in the absence of any commercial or financial relationships that could be construed as a potential conflict of interest.

## Publisher’s Note

All claims expressed in this article are solely those of the authors and do not necessarily represent those of their affiliated organizations, or those of the publisher, the editors and the reviewers. Any product that may be evaluated in this article, or claim that may be made by its manufacturer, is not guaranteed or endorsed by the publisher.

## Footnotes

1
https://bigsdb.pasteur.fr/listeria/listeria.html

2
http://www.genomicepidemiology.org/
