# Supplementary material for: Virulence and Antibiotic Resistance Genes in Listeria monocytogenes Strains Isolated From Ready-to-Eat Foods in Chile
Source: Front Microbiol. 2022 Feb 21;12:796040. doi: 10.3389/fmicb.2021.796040 (PMC8921925; doi:10.3389/fmicb.2021.796040)
Supplement: Supplementary file 1 [file Table_1.DOCX]

**Supplementary Table 1**. Detection of mobile genetics elements (MGE) in *Listeria monocytogenes* strains

| Strains ID | *ST | **CC | Insertion sequence | Accession number |
| --- | --- | --- | --- | --- |
| MRL-19-00634 | 451 | 11 | No |  |
| MRL-19-00637 | 5 | 5 | ISLmo5  ISS1N  cn_12410_ISS1N  ISLmo3 | CP020834.1  M37395  M37395  CP022021.1 |
| MRL-19-00656 | 193 | 193 | ISLmo3 | CP022021.1 |
| MRL-19-00657 | 7 | 7 | No |  |
| MRL-19-00658 | 9 | 9 | ISLmo9  ISS1N  cn_8566_ISS1N | CP006611  M37395  M37395 |
| MRL-19-00660 | 14 | 14 | No |  |
| MRL-19-00662 | 3 | 3 | ISLmo7  ISLmo9  ISLmo8 | CP006611.1  CP006611  CP006611 |
| MRL-19-00666 | 8 | 8 | No |  |
| MRL-19-00667 | 8 | 8 | ISS1N | M37395 |
| MRL-19-00670 | 8 | 8 | No |  |
| MRL-19-00672 | 2763 | 5 | ISLmo3  ISLmo5  ISLmo8 | CP022021.1  CP020834.1  CP006611 |
| MRL-19-00673 | 2763 | 5 | ISLmo8  ISLmo5  ISLmo3 | CP006611  CP020834.1  CP022021.1 |
| MRL-19-00675 | 1 | 1 | ISLmo3 | CP022021.1 |
| MRL-19-00677 | 2763 | 5 | ISLmo3  ISLmo5  ISLmo8 | CP022021.1  CP020834.1  CP006611 |

*ST: sequence Type; **CC: Clonal Complex;
