# Supplementary material for: Virulence and Antibiotic Resistance Genes in Listeria monocytogenes Strains Isolated From Ready-to-Eat Foods in Chile
Source: Front Microbiol. 2022 Feb 21;12:796040. doi: 10.3389/fmicb.2021.796040 (PMC8921925; doi:10.3389/fmicb.2021.796040)
Supplement: Supplementary file 2 [file Table_2.DOCX]

**Supplementary Table 2.** Spacer sequences that comprise the CRISPR arrays. These spacers are associated with sequences corresponding to phages.

|  | | | | | | | |
| --- | --- | --- | --- | --- | --- | --- | --- |
| **GENOME MRL-19-00634** | | | | | | | |
| **Phage ID** | **Phage Name** | **Spacer ID** | **Identity** | **Coverage** | **Hit Length** | **Hit Postition** | **Spacer Sequence** |
| ref\|NC_003216.1\| | Listeria phage A118 | 1.7\|45653\|36\|MRL-19-00634_contig5 | 97.22 | 0.94444444 | 36 | 6982-7017 | GAAATCGAGGTGGTTTGATGCCGATTAAAGTACGTG |
| ref\|NC_003291.2\| | Listeria phage 2389 | 1.1\|45265\|36\|MRL-19-00634_contig5 | 97.22 | 0.94444444 | 36 | 14057-14022 | ATATTTGACCGTGCCCGGTAAAACTACCGCAAACGT |
| ref\|NC_003291.2\| | Listeria phage 2389 | 1.14\|46108\|36\|MRL-19-00634_contig5 | 97.22 | 0.94444444 | 36 | 24855-24820 | TGATGTAATTCCTACTTTTGTCATTGTTTTAAGAGC |
| ref\|NC_003291.2\| | Listeria phage 2389 | 1.17\|46301\|37\|MRL-19-00634_contig5 | 91.89 | 0.91891892 | 37 | 20436-20400 | AAAACAAGTTCAGCATGATAGTAGTTCGTTAAAAGAA |
| ref\|NC_009810.1\| | Listeria phage A500 | 1.7\|45653\|36\|MRL-19-00634_contig5 | 94.44 | 0.94444444 | 36 | 6959-6994 | GAAATCGAGGTGGTTTGATGCCGATTAAAGTACGTG |
| ref\|NC_009812.1\| | Listeria phage B025 | 1.16\|46237\|35\|MRL-19-00634_contig5 | 100 | 0.97142857 | 33 | 1955-1923 | CAATATCTTCTATTTCATCCGCACGCCAAAGAGCA |
| ref\|NC_009812.1\| | Listeria phage B025 | 1.17\|46301\|37\|MRL-19-00634_contig5 | 100 | 0.91891892 | 37 | 25170-25134 | AAAACAAGTTCAGCATGATAGTAGTTCGTTAAAAGAA |
| ref\|NC_009812.1\| | Listeria phage B025 | 2.2\|63468\|37\|MRL-19-00634_contig5 | 100 | 0.91891892 | 36 | 3931-3896 | CTTAATTTGATAGTCATAATTTATTTCCTCCTATTTT |
| ref\|NC_009812.1\| | Listeria phage B025 | 2.7\|63789\|36\|MRL-19-00634_contig5 | 97.06 | 0.94444444 | 34 | 5078-5045 | CGCCATTTACAATCACCCTTCCGGAGTTTGATCTGT |
| ref\|NC_009813.1\| | Listeria phage B054 | 1.21\|46560\|35\|MRL-19-00634_contig5 | 100 | 0.97142857 | 35 | 39332-39298 | AGAATAGTTTCTTTTTTGTTGGACCATTTATAGGT |
| ref\|NC_009815.1\| | Listeria phage A006 | 2.1\|63404\|36\|MRL-19-00634_contig5 | 100 | 0.94444444 | 35 | 15712-15746 | CGAAATTGTAAAATCTATATCCGTTGATTCAACAAA |
| ref\|NC_018831.1\| | Listeria phage P70 | 1.24\|46755\|40\|MRL-19-00634_contig5 | 100 | 0.85 | 34 | 54942-54975 | GGGATATGTTGGATAGTAGTATAATAACTGTGCTAAAATA |
| ref\|NC_018831.1\| | Listeria phage P70 | 1.25\|46824\|34\|MRL-19-00634_contig5 | 96.55 | 1 | 29 | 52891-52919 | AACGAGTAGAAGTAATTTGGGATAAAGAAGTGAC |
| ref\|NC_021539.2\| | Listeria phage LP-030-2 | 1.1\|45265\|36\|MRL-19-00634_contig5 | 97.22 | 0.94444444 | 36 | 14079-14044 | ATATTTGACCGTGCCCGGTAAAACTACCGCAAACGT |
| ref\|NC_021539.2\| | Listeria phage LP-030-2 | 1.14\|46108\|36\|MRL-19-00634_contig5 | 97.22 | 0.94444444 | 36 | 25132-25097 | TGATGTAATTCCTACTTTTGTCATTGTTTTAAGAGC |
| ref\|NC_021539.2\| | Listeria phage LP-030-2 | 1.17\|46301\|37\|MRL-19-00634_contig5 | 91.89 | 0.91891892 | 37 | 20713-20677 | AAAACAAGTTCAGCATGATAGTAGTTCGTTAAAAGAA |
| ref\|NC_021539.2\| | Listeria phage LP-030-2 | 2.3\|63533\|36\|MRL-19-00634_contig5 | 97.22 | 0.94444444 | 36 | 18961-18926 | TATAGAAAGGAGTTGATACAGTGAAGGATGTTTTAG |
| ref\|NC_021785.1\| | Listeria phage LP-110 | 1.24\|46755\|40\|MRL-19-00634_contig5 | 100 | 0.85 | 34 | 10526-10559 | GGGATATGTTGGATAGTAGTATAATAACTGTGCTAAAATA |
| ref\|NC_021785.1\| | Listeria phage LP-110 | 1.25\|46824\|34\|MRL-19-00634_contig5 | 100 | 1 | 27 | 9180-9206 | AACGAGTAGAAGTAATTTGGGATAAAGAAGTGAC |
| ref\|NC_021787.2\| | Listeria phage LP-037 | 1.24\|46755\|40\|MRL-19-00634_contig5 | 100 | 0.85 | 34 | 5005-5038 | GGGATATGTTGGATAGTAGTATAATAACTGTGCTAAAATA |
| ref\|NC_021787.2\| | Listeria phage LP-037 | 1.25\|46824\|34\|MRL-19-00634_contig5 | 96.55 | 1 | 29 | 3641-3669 | AACGAGTAGAAGTAATTTGGGATAAAGAAGTGAC |
| ref\|NC_024375.1\| | Listeria phage LP-026 | 1.24\|46755\|40\|MRL-19-00634_contig5 | 100 | 0.85 | 35 | 31156-31190 | GGGATATGTTGGATAGTAGTATAATAACTGTGCTAAAATA |
| ref\|NC_024375.1\| | Listeria phage LP-026 | 1.25\|46824\|34\|MRL-19-00634_contig5 | 100 | 1 | 27 | 29797-29823 | AACGAGTAGAAGTAATTTGGGATAAAGAAGTGAC |
| ref\|NC_024384.1\| | Listeria phage LP-030-3 | 1.7\|45653\|36\|MRL-19-00634_contig5 | 100 | 0.94444444 | 25 | 15379-15403 | GAAATCGAGGTGGTTTGATGCCGATTAAAGTACGTG |
| ref\|NC_024384.1\| | Listeria phage LP-030-3 | 1.19\|46431\|36\|MRL-19-00634_contig5 | 100 | 0.94444444 | 33 | 40268-40236 | ACTCATTTTCTTATTGGTAATCACTGTCATGTATAG |
| ref\|NC_024387.1\| | Listeria phage LP-101 | 1.4\|45459\|34\|MRL-19-00634_contig5 | 91.18 | 1 | 34 | 20897-20864 | CATGTAGATATAGAAAAAGATGTTCGAAAAGCAC |
| ref\|NC_024387.1\| | Listeria phage LP-101 | 1.16\|46237\|35\|MRL-19-00634_contig5 | 100 | 0.97142857 | 35 | 1949-1915 | CAATATCTTCTATTTCATCCGCACGCCAAAGAGCA |
| ref\|NC_024387.1\| | Listeria phage LP-101 | 1.17\|46301\|37\|MRL-19-00634_contig5 | 100 | 0.91891892 | 37 | 24669-24633 | AAAACAAGTTCAGCATGATAGTAGTTCGTTAAAAGAA |
| ref\|NC_024387.1\| | Listeria phage LP-101 | 2.2\|63468\|37\|MRL-19-00634_contig5 | 100 | 0.91891892 | 36 | 3971-3936 | CTTAATTTGATAGTCATAATTTATTTCCTCCTATTTT |
| ref\|NC_024387.1\| | Listeria phage LP-101 | 2.3\|63533\|36\|MRL-19-00634_contig5 | 97.22 | 0.94444444 | 36 | 22917-22882 | TATAGAAAGGAGTTGATACAGTGAAGGATGTTTTAG |
| ref\|NC_024387.1\| | Listeria phage LP-101 | 2.7\|63789\|36\|MRL-19-00634_contig5 | 100 | 0.94444444 | 35 | 5118-5084 | CGCCATTTACAATCACCCTTCCGGAGTTTGATCTGT |
| ref\|NC_024392.1\| | Listeria phage LP-114 | 1.24\|46755\|40\|MRL-19-00634_contig5 | 100 | 0.85 | 34 | 38330-38363 | GGGATATGTTGGATAGTAGTATAATAACTGTGCTAAAATA |
| ref\|NC_024392.1\| | Listeria phage LP-114 | 1.25\|46824\|34\|MRL-19-00634_contig5 | 96 | 1 | 25 | 36988-37012 | AACGAGTAGAAGTAATTTGGGATAAAGAAGTGAC |
| ref\|NC_028871.1\| | Listeria phage vB_LmoS_188 | 2.2\|63468\|37\|MRL-19-00634_contig5 | 92.59 | 0.91891892 | 27 | 27444-27420 | CTTAATTTGATAGTCATAATTTATTTCCTCCTATTTT |
| ref\|NC_028929.1\| | Listeria phage vB_LmoS_293 | 1.7\|45653\|36\|MRL-19-00634_contig5 | 100 | 0.94444444 | 36 | 6980-7015 | GAAATCGAGGTGGTTTGATGCCGATTAAAGTACGTG |
| **GENOME MRL-19-00657** | | | | | | | |
| ref\|NC_009810.1\| | Listeria phage A500 | 2.2\|11611\|29\|MRL-19-00657_contig31 | 100 | 0.793103448 | 29 | 36224-36252 | TATCAGCAATTGAAACTATTAAAAATGAC |
| ref\|NC_009810.1\| | Listeria phage A500 | 4.7\|1713\|30\|MRL-19-00657_contig36 | 100 | 0.766666667 | 30 | 30271-30242 | TAGTTTTTCTTCGTCCTCTAAACGCAAGCT |
| ref\|NC_009812.1\| | Listeria phage B025 | 2.3\|11677\|29\|MRL-19-00657_contig31 | 100 | 0.793103448 | 29 | 7938-7966 | CTAGACCTAGATGCTACTAGAGTGGACGA |
| ref\|NC_009812.1\| | Listeria phage B025 | 3.2\|127\|31\|MRL-19-00657_contig36 | 100 | 0.741935484 | 28 | 2459-2486 | TGGAAATGAACGATTGTCGGCATTCACGAAT |
| ref\|NC_009813.1\| | Listeria phage B054 | 3.14\|920\|30\|MRL-19-00657_contig36 | 100 | 0.766666667 | 30 | 2110-2139 | GCGTTGAACCAGAAGGTGGGAAAGAAACAC |
| ref\|NC_009815.1\| | Listeria phage A006 | 2.1\|11545\|29\|MRL-19-00657_contig31 | 100 | 0.793103448 | 29 | 4968-4940 | CGTGATTTCTTCCTCATGCGCGCTTTTGA |
| ref\|NC_018831.1\| | Listeria phage P70 | 3.4\|260\|30\|MRL-19-00657_contig36 | 100 | 0.766666667 | 30 | 20620-20591 | GAAGAAGTCACTAGTTGGGTCAAGCAAGGC |
| ref\|NC_018831.1\| | Listeria phage P70 | 3.5\|326\|30\|MRL-19-00657_contig36 | 100 | 0.766666667 | 28 | 53145-53118 | TAGTGCCAGTAAATTCAGTTCCTCCAAAGG |
| ref\|NC_021539.2\| | Listeria phage LP-030-2 | 1.7\|14194\|35\|MRL-19-00657_contig29 | 97.14 | 0.657142857 | 35 | 18926-18960 | CTAAAACATCCTTCACTGTATCAACTCCTTTCTAT |
| ref\|NC_021781.2\| | Listeria phage LP-125 | 4.6\|1647\|30\|MRL-19-00657_contig36 | 96.67 | 0.766666667 | 30 | 1706-1735 | AGTTAGATAGATAAACAAACTAAACAAAGG |
| ref\|NC_021785.1\| | Listeria phage LP-110 | 3.4\|260\|30\|MRL-19-00657_contig36 | 100 | 0.766666667 | 30 | 43463-43434 | GAAGAAGTCACTAGTTGGGTCAAGCAAGGC |
| ref\|NC_021785.1\| | Listeria phage LP-110 | 3.5\|326\|30\|MRL-19-00657_contig36 | 100 | 0.766666667 | 28 | 9430-9403 | TAGTGCCAGTAAATTCAGTTCCTCCAAAGG |
| ref\|NC_021787.2\| | Listeria phage LP-037 | 3.4\|260\|30\|MRL-19-00657_contig36 | 92.59 | 0.766666667 | 27 | 37516-37490 | GAAGAAGTCACTAGTTGGGTCAAGCAAGGC |
| ref\|NC_021787.2\| | Listeria phage LP-037 | 3.5\|326\|30\|MRL-19-00657_contig36 | 100 | 0.766666667 | 28 | 3895-3868 | TAGTGCCAGTAAATTCAGTTCCTCCAAAGG |
| ref\|NC_024360.1\| | Listeria phage LMSP-25 | 4.6\|1647\|30\|MRL-19-00657_contig36 | 96.67 | 0.766666667 | 30 | 5592-5621 | AGTTAGATAGATAAACAAACTAAACAAAGG |
| ref\|NC_024364.1\| | Listeria phage List-36 | 4.6\|1647\|30\|MRL-19-00657_contig36 | 96.67 | 0.766666667 | 30 | 256-227 | AGTTAGATAGATAAACAAACTAAACAAAGG |
| ref\|NC_024375.1\| | Listeria phage LP-026 | 3.4\|260\|30\|MRL-19-00657_contig36 | 100 | 0.766666667 | 30 | 64666-64637 | GAAGAAGTCACTAGTTGGGTCAAGCAAGGC |
| ref\|NC_024375.1\| | Listeria phage LP-026 | 3.5\|326\|30\|MRL-19-00657_contig36 | 100 | 0.766666667 | 28 | 30047-30020 | TAGTGCCAGTAAATTCAGTTCCTCCAAAGG |
| ref\|NC_024383.1\| | Listeria phage LP-083-2 | 4.6\|1647\|30\|MRL-19-00657_contig36 | 96.67 | 0.766666667 | 30 | 133572-133601 | AGTTAGATAGATAAACAAACTAAACAAAGG |
| ref\|NC_024384.1\| | Listeria phage LP-030-3 | 2.2\|11611\|29\|MRL-19-00657_contig31 | 100 | 0.793103448 | 29 | 5779-5807 | TATCAGCAATTGAAACTATTAAAAATGAC |
| ref\|NC_024384.1\| | Listeria phage LP-030-3 | 3.6\|392\|30\|MRL-19-00657_contig36 | 100 | 0.766666667 | 30 | 18116-18145 | TGTAAATGCGTTTAAATCGATGGGAAGCGC |
| ref\|NC_024384.1\| | Listeria phage LP-030-3 | 4.7\|1713\|30\|MRL-19-00657_contig36 | 96.67 | 0.766666667 | 30 | 39840-39811 | TAGTTTTTCTTCGTCCTCTAAACGCAAGCT |
| ref\|NC_024387.1\| | Listeria phage LP-101 | 1.7\|14194\|35\|MRL-19-00657_contig29 | 97.14 | 0.657142857 | 35 | 22882-22916 | CTAAAACATCCTTCACTGTATCAACTCCTTTCTAT |
| ref\|NC_024387.1\| | Listeria phage LP-101 | 2.3\|11677\|29\|MRL-19-00657_contig31 | 96.55 | 0.793103448 | 29 | 7977-8005 | CTAGACCTAGATGCTACTAGAGTGGACGA |
| ref\|NC_024387.1\| | Listeria phage LP-101 | 2.4\|11743\|29\|MRL-19-00657_contig31 | 100 | 0.793103448 | 26 | 8731-8706 | AATGCATCTTTATATTGATTTACCTCACC |
| ref\|NC_024387.1\| | Listeria phage LP-101 | 3.1\|61\|30\|MRL-19-00657_contig36 | 100 | 0.766666667 | 27 | 8732-8706 | TAATGCATCTTTATATTGATTTACCTCACC |
| ref\|NC_024387.1\| | Listeria phage LP-101 | 3.2\|127\|31\|MRL-19-00657_contig36 | 100 | 0.741935484 | 31 | 2451-2481 | TGGAAATGAACGATTGTCGGCATTCACGAAT |
| ref\|NC_024387.1\| | Listeria phage LP-101 | 3.12\|788\|30\|MRL-19-00657_contig36 | 96.43 | 0.766666667 | 28 | 18962-18989 | TAACAGTGTATTTCTATTGCTCGGTGTGTT |
| **GENOME MRL-19-00660** | | | | | | | |
|  | | | | | | | |
| ref\|NC_003216.1\| | Listeria phage A118 | 3.11\|7847\|36\|MRL-19-00660_contig45 | 100 | 0.972222222 | 36 | 18563-18598 | CTGGTTACTCAACTGGCGACAGTAATACACCTCAAT |
| ref\|NC_003216.1\| | Listeria phage A118 | 3.15\|8104\|34\|MRL-19-00660_contig45 | 100 | 1.029411765 | 34 | 19172-19205 | AGAAGCGTTAGCGGCATTGTTCGAAAGTAATTTA |
| ref\|NC_003291.2\| | Listeria phage 2389 | 1.4\|45578\|36\|MRL-19-00660_contig23 | 97.22 | 0.972222222 | 36 | 14057-14022 | ATATTTGACCGTGCCCGGTAAAACTACCGCAAACGT |
| ref\|NC_009812.1\| | Listeria phage B025 | 3.6\|7522\|36\|MRL-19-00660_contig45 | 97.22 | 0.972222222 | 36 | 27538-27503 | CGCTTCACGTTGGTTTTTCGTAGCCCAATTGCTAAA |
| ref\|NC_009813.1\| | Listeria phage B054 | 3.18\|8297\|35\|MRL-19-00660_contig45 | 91.43 | 1 | 35 | 22420-22386 | GCTTTTTTCAACAATTTTATCACCAGATGTCATTA |
| ref\|NC_009815.1\| | Listeria phage A006 | 2.3\|8132\|34\|MRL-19-00660_contig24 | 100 | 1.029411765 | 20 | 28126-28145 | GCGGATTAGAAACGATGACACTTGCCTTAAAAAA |
| ref\|NC_021539.2\| | Listeria phage LP-030-2 | 1.4\|45578\|36\|MRL-19-00660_contig23 | 97.22 | 0.972222222 | 36 | 14079-14044 | ATATTTGACCGTGCCCGGTAAAACTACCGCAAACGT |
| ref\|NC_024387.1\| | Listeria phage LP-101 | 3.16\|8167\|36\|MRL-19-00660_contig45 | 93.55 | 0.972222222 | 31 | 32765-32795 | TATAAAATATTGCCCAATGTGCGGAAGGAGTTTGGA |
| ref\|NC_028676.1\| | Sinorhizobium phage phiM9 | 3.7\|7587\|37\|MRL-19-00660_contig45 | 100 | 0.945945946 | 20 | 19284-19265 | ACGTTGGACAACGAGGTGAGTATGATATGACTCAAAT |
| ref\|NC_028929.1\| | Listeria phage vB_LmoS_293 | 2.3\|8132\|34\|MRL-19-00660_contig24 | 100 | 1.029411765 | 20 | 30024-30043 | GCGGATTAGAAACGATGACACTTGCCTTAAAAAA |
| **GENOME MRL-19-00662** | | | | | | | |
| ref\|NC_003216.1\| | Listeria phage A118 | 2.7\|7310\|30\|MRL-19-00662_contig97 | 96.67 | 0.7 | 30 | 39285-39314 | CAAGTTGAAGATATTAACTACATTCAGACA |
| ref\|NC_003216.1\| | Listeria phage A118 | 3.1\|7970\|30\|MRL-19-00662_contig97 | 100 | 0.7 | 30 | 20088-20059 | AACTTCTACTATAATAATAACTTGTCATTA |
| ref\|NC_003216.1\| | Listeria phage A118 | 5.3\|170\|36\|MRL-19-00662_contig222 | 100 | 0.583333333 | 36 | 18598-18563 | ATTGAGGTGTATTACTGTCGCCAGTTGAGTAACCAG |
| ref\|NC_009810.1\| | Listeria phage A500 | 2.7\|7310\|30\|MRL-19-00662_contig97 | 100 | 0.7 | 30 | 37232-37261 | CAAGTTGAAGATATTAACTACATTCAGACA |
| ref\|NC_009810.1\| | Listeria phage A500 | 2.8\|7376\|30\|MRL-19-00662_contig97 | 100 | 0.7 | 30 | 28041-28012 | TTGCTAATTCTTTAGCAGTATCTAAAGTTA |
| ref\|NC_009811.2\| | Listeria virus A511 | 2.10\|7508\|30\|MRL-19-00662_contig97 | 100 | 0.7 | 30 | 129539-129568 | TAATGATGAGTTACTTGGAATTTGTACAAT |
| ref\|NC_009811.2\| | Listeria virus A511 | 2.11\|7574\|30\|MRL-19-00662_contig97 | 96.67 | 0.7 | 30 | 129021-129050 | TAGTAGTATTCTTAGCAGACAATAAAAGTA |
| ref\|NC_009811.2\| | Listeria virus A511 | 3.2\|8036\|30\|MRL-19-00662_contig97 | 100 | 0.7 | 30 | 11389-11418 | CAGAGCTGTAGACAAGTCCTTTCGTGTTAC |
| ref\|NC_009813.1\| | Listeria phage B054 | 2.6\|7244\|30\|MRL-19-00662_contig97 | 100 | 0.7 | 30 | 12781-12810 | TAAACGGCGTATTCACAGGTGAAACTGAAG |
| ref\|NC_009813.1\| | Listeria phage B054 | 5.4\|235\|36\|MRL-19-00662_contig222 | 100 | 0.583333333 | 21 | 25694-25674 | AAGTGATACAATGTGTTTTTTATTGTCGATGTGGAC |
| ref\|NC_009815.1\| | Listeria phage A006 | 2.7\|7310\|30\|MRL-19-00662_contig97 | 96.67 | 0.7 | 30 | 36580-36609 | CAAGTTGAAGATATTAACTACATTCAGACA |
| ref\|NC_009815.1\| | Listeria phage A006 | 4.2\|153\|36\|MRL-19-00662_contig178 | 100 | 0.583333333 | 36 | 16707-16672 | AATATTTAACTCAATAACCCTTGCTTGTACTGTAAT |
| ref\|NC_018831.1\| | Listeria phage P70 | 2.15\|7838\|30\|MRL-19-00662_contig97 | 100 | 0.7 | 30 | 54980-54951 | CCATATTAGCACAGTTATTATACTACTATC |
| ref\|NC_020871.1\| | Listeria phage vB_LmoM_AG20 | 2.11\|7574\|30\|MRL-19-00662_contig97 | 100 | 0.7 | 30 | 129717-129746 | TAGTAGTATTCTTAGCAGACAATAAAAGTA |
| ref\|NC_021781.2\| | Listeria phage LP-125 | 2.11\|7574\|30\|MRL-19-00662_contig97 | 100 | 0.7 | 30 | 3914-3943 | TAGTAGTATTCTTAGCAGACAATAAAAGTA |
| ref\|NC_021781.2\| | Listeria phage LP-125 | 3.2\|8036\|30\|MRL-19-00662_contig97 | 100 | 0.7 | 29 | 20005-20033 | CAGAGCTGTAGACAAGTCCTTTCGTGTTAC |
| ref\|NC_021785.1\| | Listeria phage LP-110 | 2.15\|7838\|30\|MRL-19-00662_contig97 | 100 | 0.7 | 30 | 10564-10535 | CCATATTAGCACAGTTATTATACTACTATC |
| ref\|NC_021787.2\| | Listeria phage LP-037 | 2.15\|7838\|30\|MRL-19-00662_contig97 | 100 | 0.7 | 30 | 5043-5014 | CCATATTAGCACAGTTATTATACTACTATC |
| ref\|NC_024359.1\| | Listeria phage LP-048 | 2.11\|7574\|30\|MRL-19-00662_contig97 | 96.67 | 0.7 | 30 | 132349-132378 | TAGTAGTATTCTTAGCAGACAATAAAAGTA |
| ref\|NC_024359.1\| | Listeria phage LP-048 | 3.2\|8036\|30\|MRL-19-00662_contig97 | 100 | 0.7 | 30 | 13318-13347 | CAGAGCTGTAGACAAGTCCTTTCGTGTTAC |
| ref\|NC_024360.1\| | Listeria phage LMSP-25 | 2.11\|7574\|30\|MRL-19-00662_contig97 | 100 | 0.7 | 30 | 8562-8591 | TAGTAGTATTCTTAGCAGACAATAAAAGTA |
| ref\|NC_024375.1\| | Listeria phage LP-026 | 2.15\|7838\|30\|MRL-19-00662_contig97 | 100 | 0.7 | 30 | 31195-31166 | CCATATTAGCACAGTTATTATACTACTATC |
| ref\|NC_024383.1\| | Listeria phage LP-083-2 | 2.11\|7574\|30\|MRL-19-00662_contig97 | 100 | 0.7 | 30 | 981-1010 | TAGTAGTATTCTTAGCAGACAATAAAAGTA |
| ref\|NC_024383.1\| | Listeria phage LP-083-2 | 3.2\|8036\|30\|MRL-19-00662_contig97 | 100 | 0.7 | 30 | 16671-16700 | CAGAGCTGTAGACAAGTCCTTTCGTGTTAC |
| ref\|NC_024384.1\| | Listeria phage LP-030-3 | 2.7\|7310\|30\|MRL-19-00662_contig97 | 100 | 0.7 | 30 | 6787-6816 | CAAGTTGAAGATATTAACTACATTCAGACA |
| ref\|NC_024384.1\| | Listeria phage LP-030-3 | 2.8\|7376\|30\|MRL-19-00662_contig97 | 96.67 | 0.7 | 30 | 37425-37396 | TTGCTAATTCTTTAGCAGTATCTAAAGTTA |
| ref\|NC_024387.1\| | Listeria phage LP-101 | 1.3\|38438\|36\|MRL-19-00662_contig24 | 100 | 0.583333333 | 32 | 17914-17945 | TAACTCGGCTTATTTTTTATGTCAAAAAGTAGGAAG |
| ref\|NC_024387.1\| | Listeria phage LP-101 | 4.3\|218\|37\|MRL-19-00662_contig178 | 97.3 | 0.567567568 | 37 | 9186-9222 | GCATGGGAACAATTTGATGCTCTCGGTTTAAGCATTG |
| ref\|NC_024392.1\| | Listeria phage LP-114 | 2.15\|7838\|30\|MRL-19-00662_contig97 | 100 | 0.7 | 30 | 38368-38339 | CCATATTAGCACAGTTATTATACTACTATC |
| ref\|NC_024787.1\| | Listeria phage LMTA-148 | 2.11\|7574\|30\|MRL-19-00662_contig97 | 96.67 | 0.7 | 30 | 6287-6316 | TAGTAGTATTCTTAGCAGACAATAAAAGTA |
| ref\|NC_025440.1\| | Listeria phage WIL-1 | 2.11\|7574\|30\|MRL-19-00662_contig97 | 96.67 | 0.7 | 30 | 38276-38247 | TAGTAGTATTCTTAGCAGACAATAAAAGTA |
| ref\|NC_025440.1\| | Listeria phage WIL-1 | 3.2\|8036\|30\|MRL-19-00662_contig97 | 100 | 0.7 | 30 | 20705-20676 | CAGAGCTGTAGACAAGTCCTTTCGTGTTAC |
| ref\|NC_028929.1\| | Listeria phage vB_LmoS_293 | 2.8\|7376\|30\|MRL-19-00662_contig97 | 96.43 | 0.7 | 28 | 29062-29035 | TTGCTAATTCTTTAGCAGTATCTAAAGTTA |
| **GENOME MRL-19-00666** | | | | | | | |
|  | | | | | | | |
| ref\|NC_009812.1\| | Listeria phage B025 | 1.4\|269196\|35\|MRL-19-00666_contig2 | 97.06 | 0.971428571 | 34 | 5078-5045 | GCCATTTACAATCACCCTTCCGGAGTTTGATCTGT |
| ref\|NC_009815.1\| | Listeria phage A006 | 1.1\|269003\|35\|MRL-19-00666_contig2 | 100 | 0.971428571 | 35 | 15712-15746 | GAAATTGTAAAATCTATATCCGTTGATTCAACAAA |
| ref\|NC_024387.1\| | Listeria phage LP-101 | 1.4\|269196\|35\|MRL-19-00666_contig2 | 100 | 0.971428571 | 35 | 5118-5084 | GCCATTTACAATCACCCTTCCGGAGTTTGATCTGT |
| **GENOME MRL-19-00667** | | | | | | | |
| ref\|NC_009812.1\| | Listeria phage B025 | 1.1\|12093\|35\|MRL-19-00667_contig15 | 97.06 | 1 | 34 | 5045-5078 | ACAGATCAAACTCCGGAAGGGTGATTGTAAATGGC |
| ref\|NC_009812.1\| | Listeria phage B025 | 1.6\|12413\|36\|MRL-19-00667_contig15 | 100 | 0.972222222 | 36 | 3896-3931 | AAAATAGGAGGAAATAAATTATGACTATCAAATTAA |
| ref\|NC_009815.1\| | Listeria phage A006 | 1.7\|12478\|35\|MRL-19-00667_contig15 | 100 | 1 | 35 | 15746-15712 | TTTGTTGAATCAACGGATATAGATTTTACAATTTC |
| ref\|NC_021539.2\| | Listeria phage LP-030-2 | 1.5\|12349\|35\|MRL-19-00667_contig15 | 97.14 | 1 | 35 | 18926-18960 | CTAAAACATCCTTCACTGTATCAACTCCTTTCTAT |
| ref\|NC_024387.1\| | Listeria phage LP-101 | 1.1\|12093\|35\|MRL-19-00667_contig15 | 100 | 1 | 35 | 5084-5118 | ACAGATCAAACTCCGGAAGGGTGATTGTAAATGGC |
| ref\|NC_024387.1\| | Listeria phage LP-101 | 1.5\|12349\|35\|MRL-19-00667_contig15 | 97.14 | 1 | 35 | 22882-22916 | CTAAAACATCCTTCACTGTATCAACTCCTTTCTAT |
| ref\|NC_024387.1\| | Listeria phage LP-101 | 1.6\|12413\|36\|MRL-19-00667_contig15 | 100 | 0.972222222 | 36 | 3936-3971 | AAAATAGGAGGAAATAAATTATGACTATCAAATTAA |
| ref\|NC_028871.1\| | Listeria phage vB_LmoS_188 | 1.6\|12413\|36\|MRL-19-00667_contig15 | 92.59 | 0.972222222 | 27 | 27420-27444 | AAAATAGGAGGAAATAAATTATGACTATCAAATTAA |
| **GENOME MRL-19-00670** | | | | | | | |
| ref\|NC_009812.1\| | Listeria phage B025 | 1.1\|7207\|35\|MRL-19-00670_contig125 | 97.06 | 0.971428571 | 34 | 5045-5078 | ACAGATCAAACTCCGGAAGGGTGATTGTAAATGGC |
| ref\|NC_024387.1\| | Listeria phage LP-101 | 1.1\|7207\|35\|MRL-19-00670_contig125 | 100 | 0.971428571 | 35 | 5084-5118 | ACAGATCAAACTCCGGAAGGGTGATTGTAAATGGC |
