# Supplementary material for: Virulence and Antibiotic Resistance Genes in Listeria monocytogenes Strains Isolated From Ready-to-Eat Foods in Chile
Source: Front Microbiol. 2022 Feb 21;12:796040. doi: 10.3389/fmicb.2021.796040 (PMC8921925; doi:10.3389/fmicb.2021.796040)
Supplement: Supplementary file 3 [file Table_3.DOCX]

**Supplementary Table 3.** Protospacer adjacent motifs (PAMs) associated with spacer sequences. The PAM sequences are shown in red at the beginning and end of the sequence.

| **MRL-19-00634** | | | |
| --- | --- | --- | --- |
| **No. De espaciador** | **Secuencia (3´-5´)** | **Posición** | **Phage associated** |
| 10 | **TTCAAGGT**AAGAAAATTGCTTGATGATAGTACGACTTGAACAAAA**TTATTAAA** | 46338-46302 | Listeria phage B025 |
| 10 | TTCAAGGTAAGAAAATTGCTTGATGATAGTACGACTTGAACAAAATTCTCAAA | 46338-46302 | Listeria phage LP-101 |
| 18 | TTCCGGGTAACCTAAAATAATTTATGTCTTAGTGCCTAACATAAAGGTTGAAA | 45820-45784 | PHAGE_Lister_A118 |
| 18 | TTCCGGGTAACCTAAAATAATTTATGTCTTAGTGCCTAACATAAAGGTTGAAA | 45820-45785 | PHAGE_Lister_A500 |
| 26 | TATTCGGTTGCAAACGCCATCAAAATGGCCCGTGCCAGTTTATACATTAACT | 45301-45266 | PHAGE_Lister_LP_030_2_NC_021539 |
| 20 | CTCCAGGTGTGCATGAAATTAGCCGTAGTTTGGTGGAGCTAAAGGGTTACTA | 45689-45654 | Listeria phage vB_LmoS_293 |
| 3 | TTATGTGGATAAAATCGTGTCAATAATATGATGATAGGTTGTATAGCAGGTATATT | 46795-46756 | Listeria phage LP-018 |
| 12 | TAAACGGATTTTTATCGTTTTGTGGAATAAGCTGTGGAATAAATGATCTAA | 46208-46174 | PHAGE_Lister_A006 |
| 14 | AATAGGGATGTTCACGATCTTGTCTTCAATATTAAACTTGCAATGATTTGAAG | 46079-46043 | PHAGE_Lister_B054 |
| 6 | TTTAAGGTTGGATATTTACCAGGTTGTTTTTTCTTTGATAAGATCGAAGAT | 46595-46561 | PHAGE_Lister_B054 |
| 21 | ATGATGGTTCTTCACCTTATACAACCTCACCAATTTATACTGTGCCTATATAC | 45624-45588 | **PHAGE_Lister_LP_030_2(47))** |
| 9 | AGCGCAGTCGGTAATTGCTGCGACCTTATCAATGGTGTAACGGAAAAGGT | 46402-46368 | PHAGE_Lister_A006) |
| 11 | AAACAGGTACGAGAAACCGCACGCCTACTTTATCTTCTATAACTACATCTA | 46272-46238 | Listeria phage LP-HM00113468 |
| 11 | AAACAGGTACGAGAAACCGCACGCCTACTTTATCTTCTATAACTACATCTA | 46272-46239 | KJ094023 Listeria phage LP-101 |
| 18 | TTCCGGGTAACCTAAAATAATTTATGTCTCAGTGCCTAACATAAAGGTTGAAA | 45820-45784 | PHAGE_Lister_A500 |
| 26 | TATTCGGTTGCAAGCGCCATCAAAATGGCCCGTGCCAGTTTATACATTAACT | 45301-45266 | JX120799 Listeria phage LP-030-2 |
| 8 | TGCTAGGTGAAATGTACTGTCACTAATGGTTATTCTTTTACTCATCGCGCGT | 40233-40268 | KJ094022 Listeria phage LP-030-3 |
| 13 | TGACCGGTCGAGAATTTTGTTACTGTTTTCATCCGTAATGTAGTCTTTTCTT | 24820-24855 | AJ312240 Bacteriophage PSA complete genome |
| 3 | TTATGTGGGTATAATCGTGTCAATAATATGATGATAGGTTGTATAGGCGGTATATT | 47660-47623 | MN128593 Listeria phage LP-031 |
| 24 | GCCCTGGTTATAATGATAACCTTTTTCGCGCTAGACAGTGTTAATGTAATAAG | 45430-45394 | PHAGE_Lister_A006 |
| **MRL-19-00656** | | | |
| 3 | TACATTTAAACCAAGGAAACCTTTTGTTGTTATTGCACATCATAACAAAATAA | 474086-474122 | Listeria monocytogenes strain NCTC7974 plasmid 5 |
| 4 | AACATTTACGTCTTCGTCGTTTTATTGGTTTTCATTACTGAAAGTAAAATTA | 35323-35358 | Listeria monocytogenes strain FDAARGOS_57 plasmid unnamed |
| 2 | ATAGAGGTTGCTATTATCCTATAAAACCTAAACGGACGCAAGGAAATCTTCG | 35191-35226 | PHAGE_Lister_A006(7)) |
